# Feasibility of Noninvasive Diagnosis of Spinal Vascular Diseases Using Time-Resolved Angiography With Stochastic Trajectories

**DOI:** 10.3389/fneur.2021.759099

**Published:** 2021-10-14

**Authors:** Xiao-Er Wei, Ming-Hua Li, Rui-Hua Qiao, Wei-Bin Yu, Yue-Hua Li

**Affiliations:** Institute of Diagnostic and Interventional Radiology, Shanghai Jiao Tong University Affiliated Sixth People's Hospital, Shanghai, China

**Keywords:** MR angiography, spinal dural arteriovenous fistula, perimedullary arteriovenous fistula, Adamkiewicz artery, time-resolved

## Abstract

**Background and Purpose:** To determine the feasibility of time-resolved angiography with stochastic trajectories (TWIST) in the diagnosis of spinal dural arteriovenous fistula (SDAVF) and perimedullary arteriovenous fistula (PAVF).

**Methods:** A total of 11 negative patients with TWIST examination were retrospective analyzed and then 18 patients with suspected spinal vascular diseases underwent TWIST. For negative patients, Adamkiewicz artery (AKA), great anterior radiculomedullary vein (GARV) and anterior spinal artery (ASA) were retrospective analyzed. In patients, the results of TWIST were compared with those of DSA.

**Results:** The displaying rates of the ASA, AKA and GARV in 11 negative patients were 100, 90.9, and 90.9%, respectively. The AKA and GARV were separated on TWIST. Of 18 patients, 11 and three were diagnosed with SDAVF and PAVF, respectively. The spinal cord vascular malformation diagnosed on TWIST was consistent with DSA with an excellent intermodality agreement (Kappa = 0.92, *p* < 0.001). The feeding artery and side of all 11 SDAVF patients were displayed on TWIST and the results were consistent with DSA. For PAVF patients, the feeding artery in two patients and the sides as displayed on TWIST were consistent with DSA.

**Conclusions:** TWIST enables the differentiation of the spinal artery and vein and the differential diagnosis of SDAVF and PAVF.

## Introduction

Spinal vascular malformations are rare, and its diagnosis may be challenging on conventional MRI (1). Digital subtraction angiography (DSA) is the gold standard in the diagnosis of spinal vascular diseases and has superior spatial and temporal resolution to display the fistulas, feeding arteries, and draining veins (2). However, DSA is an invasive and time-consuming technique associated with high radiation doses, a large amount of contrast media, and several severe complications (3).

Three-dimensional contrast-enhanced magnetic resonance angiography (3D CE-MRA) has been used in the diagnosis of spinal vascular diseases. With the technique, high-resolution images are obtained, and spinal vessels are clearly displayed. The dynamic aspect of the technique enables the normal vessels and vascular malformation of the spinal cord to be clearly displayed (4–6). However, the temporal resolution of conventional CE-MRA is longer than the spinal circulation time, which may be about 10 s (7–9). On conventional CE-MRA, the artery, and the vein of the spinal cord may begin to develop at the same phase. Thus, it is difficult to differentiate the feeding artery from the tortuous vessels in patients with spinal vascular disease.

Time-resolved angiography with interleaved stochastic trajectories (TWIST) is a time-resolved MRA technique, and high temporal and spatial resolutions can be achieved. This technique is based with frequent sampling of central k-space data and view sharing for the peripheral k-space of dynamic phases. TWIST provides a non-invasive time-resolved angiographic dataset with detailed temporal information (10–12). It was initially used in the evaluation of peripheral, abdominal, and thoracic blood vessels (12). As a time-resolved MRA technique, TWIST is a sequence that balances the temporal resolution and the spatial resolution, and the temporal resolution is significantly improved compared with that of conventional CE-MRA (13, 14). TWIST is therefore considered to have certain advantages in differentiating between the spinal artery and vein. In spinal dural arteriovenous fistula (SDAVF), the feeding artery and the drainage vein communicated at the level of the intervertebral foramen. However, in perimedullary arteriovenous fistula (PAVF), the feeding artery and the drainage vein communicated directly at the spinal cord surface. Therefore, it may be useful to demonstrate the feeding artery and fistula for the differentiation of SDAVF from PAVF.

So, the aims of this study are as follows: first, to test the temporal and spatial resolution of TWIST, wherein we use TWIST to differentiate between the AKA, GARV, and ASA, and second, to investigate the value of TWIST in the diagnosis of spinal vessel malformation (SDAVF and PAVF) and the prediction of feeding arteries and fistulas.

## Materials and Methods

### Patients

The study was approved by the Institutional Review Board of our hospital and performed in accordance with the Declaration of Helsinki. Written informed consent was obtained from all patients.

Between October 2015 and March 2016, we retrospectively analyzed the data of patients who underwent TWIST examination. The inclusion criteria were as follows: (1) no abnormality in the spinal cord on conventional magnetic resonance imaging (MRI), (2) no void signal in the spinal canal on conventional MRI, and (3) no abnormality on TWIST. A total of 11 patients with negative findings were included in this study.

Between June 2016 and October 2017, patients with suspected spinal vascular malformations were included in our study for TWIST examination followed by conventional DSA. The inclusion criteria were (1) spinal cord edema on conventional MRI, (2) flow void signal around the spinal cord on conventional MRI, and (3) clinical symptoms consistent with spinal vascular malformation. Patients with a history of treated spinal vascular malformation were excluded. The time interval between TWIST and DSA was 1–6 days.

### MRI

MRI examination was performed on a 3T MR scanner (MAGENTOM, Verio, Siemens Healthcare, Erlangen, Germany). A T2-weighted fast spin-echo with fat-suppression sequence was performed to visualize the anatomy of the spine and localize the vertebrae, which served as a reference to determine the level of the segmental artery connected to the AKA and feeding artery. The acquisition parameters were as follows: repetition time/echo time (TR/TE), 4,000/53 ms; inversion time, 200 ms; flip angle, 150°. The craniocaudal field of view (FOV) was 440 mm × 440 mm, the number of sagittal slices was 13, and the slice thickness was 3.5 mm. The measured voxel size was 1.7 mm × 1.0 mm × 3.5 mm.

TWIST was performed with an RF spoiled 3D fast low-angle shot (FLASH) gradient recalled echo sequence with interleaved stochastic trajectories, and the parameters were as follows: TR/TE, 3.28/1.23 ms; band width, 660 Hz; flip angle, 14°; parallel imaging by GRAPPA with an acceleration factor *R* = 3 and 24 reference lines along the phase encoding direction; partial Fourier imaging 6/8 along the phase and slice encoding direction; matrix size, 448 × 362.8; rectangle FOV, 400 × 262.4 to 400 × 320 mm; effective voxel size, 0.9 mm × 0.9 mm × 0.6 mm; echo-sharing density (central region A = 15%, peripheral region B = 20%); and temporal resolution, 7.2 s. Fifteen continuous acquisitions were performed. The contrast agent injection was started together with the second acquisition of the time-resolved MRA recording. The initial acquisitions were used to reach the steady state and to obtain a precontrast series for image subtraction.

### DSA

Conventional DSA was performed on a monoplanar unit (Axiom Artis VB22N, Siemens Healthcare, Erlangen, Germany). Based on the results of TWIST, Seldinger technology by femoral artery puncture was used, and selective arterial injections with iodinated contrast agent (Omnipaque 300 mg/ml; GE Healthcare, Shanghai, China) were performed to identify the type of spinal vascular malformation, feeding artery, level, side, and the fistula. If necessary, additional injections were made into both vertebral arteries, the costcervical arteries, the thyrocervical trunks, and the iliolumbar arteries.

### Image Processing and Review

All images were analyzed on the Siemens workstation. For the negative patients, curved planar reformation and maximum intensity projection (MIP) were used to identify the ASA, AKA, and GARV. First, MIP images were reformatted in sagittal view. The curved paths on the anterior and posterior surfaces of the spinal cord were drawn on these MIP images. Reformation resulted in a curved coronal plane MIP image in which the midline vessels were shown in the midline. Usually, the ASA was located at the midline of the anterior cord surface. The AKA was defined as the hairpin-shaped vessel ascending from the neural foramen to the anterior cord surface. GARV was developed later than the AKA and had a larger caliber from the anterior cord surface to the neuronal foramen. The level of origin of the AKA was defined as the vertebral level according to the sagittal fat-suppression T2W images. For patients, MRA images were analyzed before the DSA examination. The type of spinal vascular malformation, feeding arteries, level, side, and the length of the draining vein were evaluated by curved planar reformation, MIP, and multiple planar reconstruction. All these decisions were made by consensus between the two neuroradiologist observers.

### Statistical Analysis

SPSS for Windows software (version 16.0; Chicago, IL, USA) was used for statistical analysis, with *p* < 0.05 considered significant. The level of interobserver agreement (between observers 1 and 2 for TWIST) in negative patients with respect to the level and side of the AKA and GARV and with respect to the display results of ASA was determined by calculating the Kappa value (0.20, poor; 0.21–0.40, fair; 0.41–0.60, moderate; 0.61–0.80, good; 0.81–0.90, very good; and >0.90, excellent agreement). The level of interobserver agreement (between observers 1 and 2 for TWIST) in patients with suspected spinal vascular malformations was also evaluated by the Kappa value. In addition, the intermodality agreement between TWIST and DSA in patients with spinal vascular malformations was also evaluated by the Kappa value.

## Results

**Figure 1:**
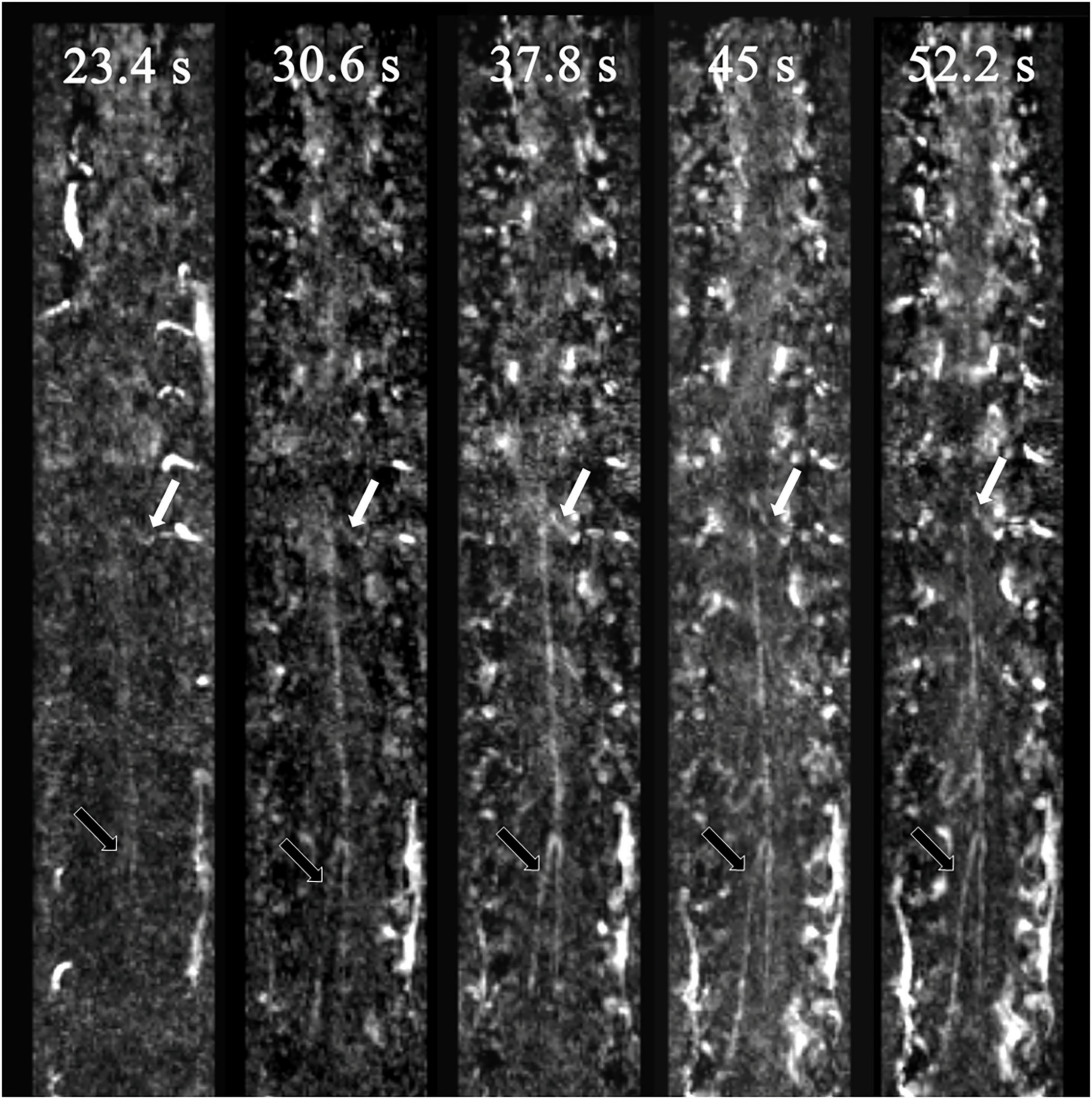
Spinal vessels of a negative patient. The white arrows indicate the AKA, and the black arrows indicate the GARV. AKA enhancement occurred one phase earlier than that for the GARV. Therefore, the AKA and GARV were dynamically separated.

All 11 negative patients (seven men; mean age, 46.8 ± 41.1 years) were included in the final analysis. Both observers agreed on the AKA and GARV, with the interobserver agreement that was very good (Kappa = 0.885, *p* < 0.001; kappa = 0.874, and *p* < 0.001, respectively). The difference in display rate of ASA between observer 1 and 2 was not significant (*p* > 0.05; Table 1). The ASA was displayed in all of the 11 negative patients. The AKA and GARV were shown in 10 (90.9%) negative patients. Of these 10 negative patients, the AKA originated from the left-sided segmental artery in eight volunteers (80%), with the segmental origin level localized between T7 and L2. For GARV, its segmental drainage level was between L1 and L3 in seven volunteers (70%) with right-sided drainage (Table 2; Figure 1).

**Table 1:** The interobserver agreement of AKA and GARV and the display rate of ASA on time-resolved angiography with stochastic trajectories between two observers.

**Case number**	**AKA**		**ASA**		**GARV**	
	**Observer 1**	**Observer 2**	**Observer 1**	**Observer 2**	**Observer 1**	**Observer 2**
	**Level/side**	**Level/side**	**Display**	**Display**	**Level/side**	**Level/side**
(1)	T7/left	T7/left	Yes	Yes	L1/right	L1/right
(2)	T8/left	T8/left	Yes	Yes	L2/left	L2/left
(3)	T8/right	T7/right	Yes	Yes	L3/right	L3/right
(4)	L1/left	L1/left	Yes	Yes	L1/right	L1/right
(5)	T12/left	T12/left	Yes	Yes	L2/right	L2/right
(6)	T11/right	T11/right	Yes	Yes	L3/left	L3/left
(7)	L2/left	L2/left	Yes	Yes	No	T12/right
(8)	T9/left	T9/left	Yes	Yes	L3/left	L3/left
(9)	T10/left	T10/left	Yes	Yes	L2/right	L2/right
(10)	T9/left	T9/left	Yes	Yes	L1/right	L1/right
(11)	–	–	Yes	No	L1/right	L1/right

*The interobserver agreement of AKA between observers 1 and 2 was very good (kappa = 0.885, p < 0.001). The interobserver agreement of GARV between observers 1 and 2 was also very good (kappa = 0.874, p < 0.001). For ASA, the difference of display rate between observers 1 and 2 was not significant (p > 0.05)*.

**Table 2:** The characteristics of AKA, ASA, and GARV in negative patients on time-resolved angiography with stochastic trajectories.

**Case**	**Sex/age**	**AKA**		**ASA display**	**GARV**	
		**Level**	**Side**		**Level**	**Side**
(1)	M/34	T7	Left	Yes	L1	Right
(2)	M/57	T8	Left	Yes	L2	Left
(3)	M/23	T8	Right	Yes	L3	Right
(4)	M/41	L1	Left	Yes	L1	Right
(5)	M/66	T12	Left	Yes	L2	Right
(6)	M/54	T11	Right	Yes	L3	Left
(7)	M/38	L2	Left	Yes	No	No
(8)	F/49	T9	Left	Yes	L3	Left
(9)	F/64	T10	Left	Yes	L2	Right
(10)	F/32	T9	Left	Yes	L1	Right
(11)	F/57	–	–	Yes	L1	Right

**Figure 2:**
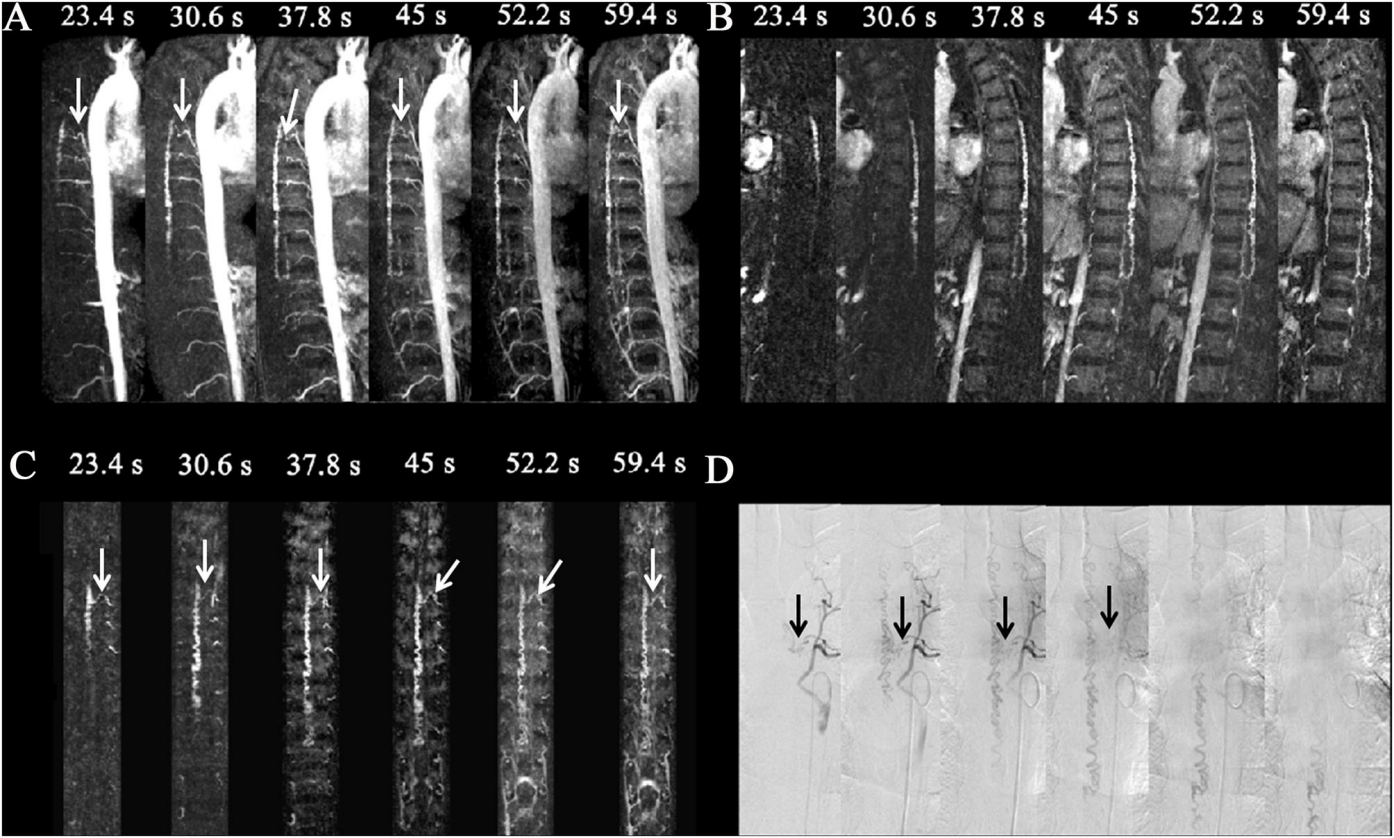
Spinal dural arteriovenous fistula (SDAVF) in a 46-year-old patient. Comparison of visualization capabilities of magnetic resonance angiography and digital subtraction angiography (DSA). **(A)** The dynamic sagittal maximum intensity projections (MIPs) of the time-resolved angiography with stochastic trajectories (TWIST) examination. The feeding artery and fistula (T6, white arrows) were displayed clearly, and the length of the dilated vein was gradually expanded. **(B)** The dynamic target sagittal MIPs of the TWIST examination. The length of the dilated vein was clearly displayed. **(C)** The dynamic target coronal curved planar reformation MIPs of the TWIST examination. The feeding artery and fistula (T6, left, white arrows) were displayed clearly. **(D)** DSA was performed, which confirmed the diagnosis of SDAVF with a level of the feeding artery of T6 (left, black arrows).

**Figure 3:**
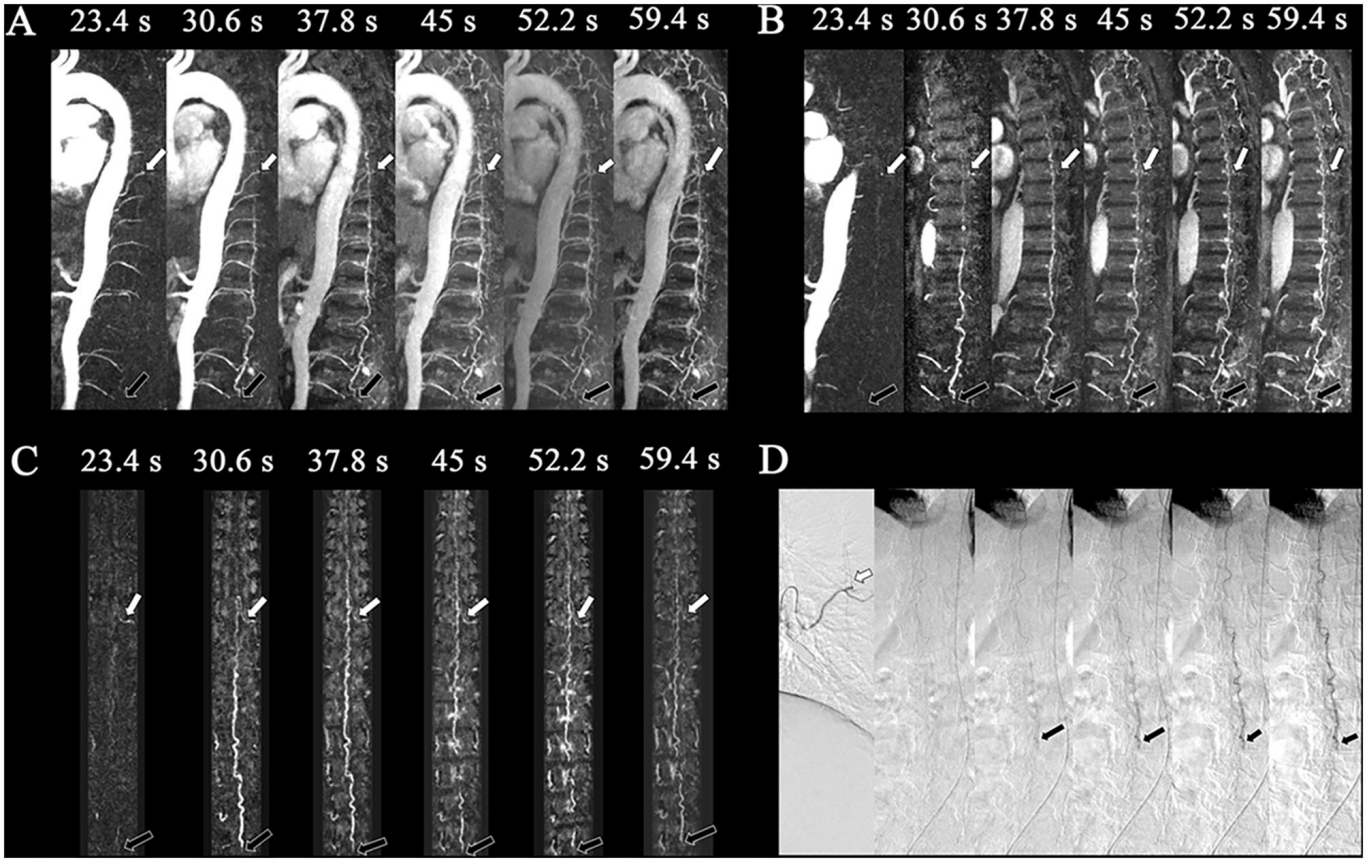
Perimedullary arteriovenous fistula (PAVF) in a 58-year-old patient. Comparison of the visualization capabilities of magnetic resonance angiography and digital subtraction angiography (DSA). **(A)** The dynamic sagittal maximum intensity projections (MIPs) of the time-resolved angiography with stochastic trajectories (TWIST) examination. In the first phase, the feeding artery that originated from T8 (white arrows) was clearly shown, and then the drainage vein was developed on the following phase with the fistula (L4, black arrows). The length of the dilated vein was also displayed. **(B)** The dynamic target sagittal MIPs of the TWIST examination. In the first phase, the feeding artery that originated from T8 (white arrows) was clearly shown, and then the drainage vein was developed on the following phase with the fistula (L4, black arrows). **(C)** The dynamic target coronal curved planar reformation MIPs of the TWIST examination. The feeding artery (T8, left, white arrows) and fistula (L4, black arrows) were displayed clearly. **(D)** DSA was performed, which confirmed the diagnosis of perimedullary arteriovenous fistula with the feeding arteries that originated from the intercostal artery of T8 (white arrows) and fistula located at L4 (black arrows).

Eighteen patients (11 men; mean age, 38.8 ± 15.3 years) were included in the study and underwent TWIST examination. Of these 18 patients, four patients were excluded in the final analysis due to normal appearance on both TWIST and DSA. For the other 14 patients (nine men; mean age, 40.4 ± 16.7), 11 patients were diagnosed as SDAVF, and three patients were diagnosed as PAVF. Both observers agreed on the type of spinal cord vascular malformation and the location of feeding arteries, with interobserver agreement that was excellent (Kappa = 0.92, *p* < 0.001; Table 3). The spinal cord vascular malformation diagnosed on TWIST was consistent with DSA, with an excellent intermodality agreement (Kappa = 0.92, *p* < 0.001; Table 4). The feeding artery and side of all 11 SDAVF were clearly shown on TWIST, and the results were consistent with the DSA findings (Figure 2). For three PAVF patients, the sides showing on TWIST were consistent with the DSA findings, and the feeding arteries in two patients were correctly displayed (Figure 3).

**Table 3:** The interobserver agreement of the spinal cord vascular malformations on time-resolved angiography with stochastic trajectories between two observers.

**Case number**	**Observer 1**			**Observer 2**			**Interobserver agreement**
	**Disease type**	**Feeding arteries**	**Side**	**Disease type**	**Feeding arteries**	**Side**	
(1)	SDAVF	T10	Left	SDAVF	T10	Left	Kappa = 0.92, *p* < 0.001
(2)	SDAVF	T6	Left	SDAVF	T6	Left	
(3)	SDAVF	T6	Right	SDAVF	T6	Right	
(4)	SDAVF	T9	Left	SDAVF	T9	Left	
(5)	SDAVF	T8	Right	SDAVF	T8	Right	
(6)	SDAVF	T12	Left	SDAVF	T12	Left	
(7)	SDAVF	L1	Left	SDAVF	L1	Left	
(8)	SDAVF	T12	Left	SDAVF	T12	Left	
(9)	SDAVF	T11	Left	SDAVF	T11	Left	
(10)	SDAVF	T7	Right	SDAVF	T7	Right	
(11)	SDAVF	T12	Left	SDAVF	T12	Left	
(12)	SDAVF	T11	Left	PAVF	L3	Left	
(13)	PAVF	T8	Left	PAVF	T8	Left	
(14)	PAVF	T8/T9	Right	PAVF	T8/T9	Right	

**Table 4:** Comparison of time-resolved angiography with stochastic trajectories and digital subtraction angiography (DSA) in patients with spinal dural arteriovenous fistula (SDAVF) and perimedullary arteriovenous fistula (PAVF).

**Case**	**Sex/age**	**Disease type**	**DSA findings**		**MRA findings**		
			**Feeding arteries**	**Side**	**Feeding arteries**	**Side**	**Drain vein range**
(1)	M/32	SDAVF	T10	Left	T10	Left	9
(2)	M/46	SDAVF	T6	Left	T6	Left	10
(3)	M/65	SDAVF	T6	Right	T6	Right	6
(4)	M/18	SDAVF	T9	Left	T9	Left	7
(5)	M/56	SDAVF	T8	Right	T8	Right	7
(6)	M/71	SDAVF	T12	Left	T12	Left	9
(7)	M/28	SDAVF	L1	Left	L1	Left	5
(8)	F/42	SDAVF	T12	Left	T12	Left	6
(9)	F/37	SDAVF	T11	Left	T11	Left	7
(10)	F/21	SDAVF	T7	Right	T7	Right	4
(11)	F/32	SDAVF	T12	Left	T12	Left	6
(12)	M/35	PAVF	L3	Left	T11	Left	8
(13)	M/58	PAVF	T8	Left	T8	Left	9
(14)	F/24	PAVF	T8/T9	Right	T8/T9	Right	11

## Discussion

In this study, to test the temporal and spatial resolution of TWIST, we use it to differentiate between the AKA, GARV, and ASA. The time resolution and spatial resolution of CE-MRA are two factors that interact with each other (15). To obtain good spatial resolution, a long time is required for scanning, which results in a reduction in temporal resolution. However, to improve the temporal resolution, the spatial resolution must be sacrificed, resulting in a decline in the quality of images. How to balance these two key parameters is, therefore, the key to spinal CE-MRA application.

Although some studies have differentiated between the AKA and GARV on the basis of the vessel signal intensity and its changing trend, the signal intensity was correlated with the diameter of the vessel rather than its arteriovenous characteristics because the spinal vessel was tiny (16). Thus, certain limitations exist when discriminating between the spinal artery and vein on conventional CE-MRA due to its lower temporal resolution.

In this study, at first, to guarantee the spatial resolution, the ability to display the AKA was used as the reference. The diameter of the AKA was 0.5–1.0 mm (2). The spatial resolution of TWIST in our study was 0.9 mm × 0.9 mm × 0.6 mm, and the AKA was visible in 90.9% of volunteers. Meanwhile, the GARV was also visible in 90.9% of negative patients. The spatial resolution of TWIST was therefore sufficient to display the spinal vessels. On the basis of this spatial resolution, a temporal resolution of 7.2 s was reached by adjusting the filling rate of k-space and FOV. The result was that the ASA, AKA, and GARV could be displayed on TWIST, and more importantly, the AKA and GARV were separated on the same phase. To display the feeding artery and discriminate the artery and vein at the level of the feeding artery is the basis of differentiating between SDAVF and PAVF. In SDAVF, the tortuous and engorged vessels of the spinal cord surface are the drainage veins that communicate with the feeding artery at the level of the intervertebral foramen. However, in PAVF, the tortuous and engorged vessels of the spinal cord surface are the feeding artery and drainage vein. In conventional CE-MRA, the actual fistula or resolved arterial and venous phases could not be demonstrated, so the differentiation of the fistula and draining radiculomedullary vein from the engorged coronal venous plexus was difficult (1). Thus, the differentiation of SDAVF and PAVF is difficult on conventional CE-MRA. In this study, SDAVF and PAVF were differentiated on TWIST because the feeding artery and drainage vein were clearly separated in 92.9% (13/14) patients.

In this study, the sampling rate of the A zone was 15% and of the B zone was 20%, and the acceleration factor reached 3.125. Combined with GRAPPA, the temporal resolution of TWIST was 7.2 s, while it may exceed 36 s on conventional CE-MRA. The reason in obtaining this high temporal resolution was the distinct coverage of k-space during the acquisition process. In this study, the filling technique of k-space was the s-modified k-space undersampling technique (17), that is to say that the data points were divided into A and B zones. A zone was the central area with low frequency and determined the overall image contrast, and B zone was the peripheral region with higher frequency that reflected the anatomic details. The number of data points within the k-space determines the spatial resolution of the phase encoding direction and FOV within the plane and between the planes. If the number of data points in the A zone was larger, the image contrast resolution was higher, whereas if the number of data points in the B zone was larger, the spatial resolution was higher. The more the data points, the longer the acquisition time. Thus, the selection of A zone 15% and B zone 20% was made to balance the spatial resolution and temporal resolution to a certain extent.

The use of TWIST could improve the temporal resolution and the speed of scanning and have some other advantages. First, in a previous study, the level of fistula was inferred by tracing an engorged medullary vein in a retrograde direction to the neural foramen without the need to distinguish the fistula and the veins draining away from the coronal venous plexus (18). In this study, the good temporal resolution of TWIST made the post-processing simplified because the drainage vein was not fully displayed on the early phase. Second, the test-bolus technique was used in conventional CE-MRA during the injection of the contrast (8, 19). However, the start time of the scan after the injection of the contrast is still controversial because the size and the hemodynamics of the fistula in each patient may be different. In this study, the contrast was injected after the first acquisition scan to reduce human interference. Thus, TWIST simplified the operation process due to its high temporal resolution. Lastly, invasive spinal angiography requires injection into both vertebral arteries, the costocervical arteries, the thyrocervical trunks, the intercostal artery, and the iliolumbar arteries and visualization of the AKA in order to avoid a misdiagnosis. To use TWIST, the 11 SDAVF and three PAVF patients were all differentiated, and the feeding artery was visualized in 13 patients (92.9%). The results of TWIST could, therefore, be used as guidance to DSA, and the exposure time and the dosage of the contrast medium may be decreased (20).

There are some limitations of this study. First, the small sample and the variability of the hemodynamics of spinal vascular diseases may lead to some bias. Large sample studies are needed to confirm our results. Another limitation was the lower spatial and temporal resolution of TWIST compared with DSA. Therefore, DSA is still the gold standard for spinal vascular diseases. However, the advantage of TWIST is the guidance for DSA and the ability to shorten the exposure time and reduce the dosage of the contrast medium (2).

## Conclusions

Good spatial and temporal resolution images could be obtained with TWIST. As a new technique, it enabled the spinal artery and vein to be differentiated and the type, level, and feeding artery of the spinal vascular disease to be determined. TWIST could, therefore, be used in patients with spinal cord vascular diseases and provide more accurate information for the clinician.

## Data Availability Statement

The raw data supporting the conclusions of this article will be made available by the authors, without undue reservation.

## Ethics Statement

The studies involving human participants were reviewed and approved by the Institutional Review Board of Shanghai Jiao Tong University Affiliated Sixth People's Hospital. The patients/participants provided their written informed consent to participate in this study.

## Author Contributions

X-EW and Y-HL conceived and designed of the study. X-EW, R-HQ, and W-BY organized the database and performed the MRI scan. DSA was performed by M-HL. X-EW wrote the first draft of the manuscript. Y-HL and M-HL reviewed and revised the manuscript. All authors have read and approved the submitted version.

## Funding

This study was supported by the National Key Research and Development Program of China (Grant No. 2019YFC0117703), Shanghai Health and Family Planning Commission research projects (Grant No. 20164Y0065), Shanghai Municipal Education Commission-Gaofeng Clinical Medicine Grant Support (No. 2016427), the National Natural Scientific Fund of China (Nos. 81671673 and 81871329), the New Developing and Frontier Technologies of Shanghai Shen Kang Hospital Development Center (Grant No. SHDC12018117), Youth Medical Talents-Medical Imaging Practitioner Program (2018), Shanghai Key Discipline of Medical Imaging (No. 2017ZZ02005), and the Excellent Discipline Leader of Shanghai Municipal Planning Commission (Grant No. 2017BR041).

## Conflict of Interest

The authors declare that the research was conducted in the absence of any commercial or financial relationships that could be construed as a potential conflict of interest.

## Publisher's Note

All claims expressed in this article are solely those of the authors and do not necessarily represent those of their affiliated organizations, or those of the publisher, the editors and the reviewers. Any product that may be evaluated in this article, or claim that may be made by its manufacturer, is not guaranteed or endorsed by the publisher.

## References

[1] Amarouche M Hart JL Siddiqui A Hampton T Walsh DC. Time-resolved contrast-enhanced MR angiography of spinal vascular malformations. AJNR Am J Neuroradiol. (2015) 36:417–22. 10.3174/ajnr.A4164 25395661 PMC7965675

[2] Backes WH Nijenhuis RJ. Advances in spinal cord MR angiography. AJNR Am J Neuroradiol. (2008) 29:619–31. 10.3174/ajnr.A0910 18202236 PMC7978199

[3] Spampinato MV Nguyen SA Rumboldt Z. Comparison of gadobenate dimeglumine and gadodiamide in the evaluation of spinal vascular anatomy with MR angiography. AJNR Am J Neuroradiol. (2010) 31:1151–6. 10.3174/ajnr.A1974 20053811 PMC7963945

[4] Nijenhuis RJ Mull M Wilmink JT on AK Backes WH. MR angiography of the great anterior radiculomedullary artery (Adamkiewicz artery) validated by digital subtraction angiography. AJNR Am J Neuroradiol. (2006) 27:1565–72. http://www.ajnr.org/content/27/7/1565.long 16908582 PMC7977559

[5] Lindenholz A TerBrugge KG van Dijk JM Farb RI. The accuracy and utility of contrast-enhanced MR angiography for localization of spinal dural arteriovenous fistulas: the Toronto experience. Eur Radiol. (2014) 24:2885–94. 10.1007/s00330-014-3307-6 25015136

[6] Vargas MI Nguyen D Viallon M Kulcsár Z Tessitore E Rilliet B. Dynamic MR angiography (MRA) of spinal vascular diseases at 3T. Eur Radiol. (2010) 20:2491–5. 10.1007/s00330-010-1815-6 20473612

[7] Bowen BC DePrima S Pattany PM Marcillo A Madsen P Quencer RM. MR angiography of normal intradural vessels of the thoracolumbar spine. AJNR Am J Neuroradiol. (1996) 17:483–94. 8881243 PMC8337981

[8] Cao JB Cui LL Jiang XY Gao SJ Sun WG Xu K. Clinical application and diagnostic value of noninvasive spinal angiography in spinal vascular malformations. J Comput Assist Tomogr. (2014) 38:474–9. 10.1097/RCT.0b013e3182ab3ab6 24681867

[9] Oda S Utsunomiya D Hirai T Kai Y Ohmori Y Shigematsu Y. Comparison of dynamic contrast-enhanced 3T MR and 64-row multidetector CT angiography for the localization of spinal dural arteriovenous fistulas. AJNR Am J Neuroradiol. (2014) 15:407–12. 10.3174/ajnr.A3660 23907244 PMC7965769

[10] Yokota Y Fushimi Y Okada T Fujimoto K Oshima S Nakajima S. Evaluation of image quality of pituitary dynamic contrast-enhanced MRI using time-resolved angiography with interleaved stochastic trajectories (TWIST) and iterative reconstruction TWIST (IT-TWIST). J Magn Reson Imaging. (2020) 51:1497–506. 10.1002/jmri.26962 31625655

[11] Higgins LJ Koshy J Mitchell SE Weiss CR Carson KA Huisman TA. Time-resolved contrast-enhanced MRA (TWIST) with Gadofosveset trisodium in the classification of soft-tissue vascular anomalies in the head and neck in children following updated 2014 ISSVA classification: first report on systematic evaluation of MRI and TWIST in a cohort of 47 children. Clin Radiol. (2016) 71:32–9. 10.1016/j.crad.2015.09.006 26474946

[12] Sugrue G Cradock A McGee A McEntee C Eustace SK Fitzpatrick P. Subtraction of time-resolved magnetic resonance angiography images improves visualization of the pulmonary veins and left atrium in adults with congenital heart disease: a novel post-processing technique. Int J Cardiovasc Imaging. (2019) 35:1339–46. 10.1007/s10554-019-01585-x 30949869

[13] Winterer JT Blanke P Schaefer A Pache G Langer M Markl M. Bilateral contrast-enhanced MR angiography of the hand: diagnostic image quality of accelerated MRI using echo sharing with interleaved stochastic trajectories (TWIST). Eur Radiol. (2011) 21:1026–33. 10.1007/s00330-010-2002-5 21085967

[14] Davenport MS Heye T Dale BM Horvath JJ Breault SR Feuerlein S. Inter- and intra-rater reproducibility of quantitative dynamic contrast enhanced MRI using TWIST perfusion data in a uterine fibroid model. J Magn Reson Imaging. (2013) 38:329–35. 10.1002/jmri.23974 23239041

[15] Condette-Auliac S Boulin A Roccatagliata L Coskun O Guieu S Guedin P. MRI and MRA of spinal cord arteriovenous shunts. J Magn Reson Imaging. (2014) 40:1253–66. 10.1002/jmri.24591 24591106

[16] Jaspers K Nijenhuis RJ Backes WH. Differentiation of spinal cord arteries and veins by time-resolved MR angiography. J Magn Reson Imaging. (2007) 26:31–40. 10.1002/jmri.20940 17659566

[17] Vogt FM Theysohn JM Michna D Hunold P Neudorf U Kinner S. Contrast-enhanced time-resolved 4D MRA of congenital heart vessel anomalies: image quality diagnostic value compared with 3D MRA. Eur Radiol. (2013) 23:2392–404. 10.1007/s00330-013-2845-7 23645330

[18] Luetmer PH Lane JI Gilbertson JR Bernstein MA Huston J 3rd Atkinson JL. Preangiographic evaluation of spinal dural arteriovenous fistulas with elliptic centric contrast-enhanced MR Angiography and effect on radiation dose and volume of iodinated contrast material. AJNR Am J Neuroradiol. (2005) 26:711–8. Available online at: http://www.ajnr.org/content/26/4/711.lon 15814910 PMC7977123

[19] Mull M Nijenhuis RJ Backes WH Krings T Wilmink JT Thron A. Value and limitations of contrast-enhanced MR angiography in spinal arteriovenous malformations and dural arteriovenous fistulas. AJNR Am J Neuroradiol. (2007) 28:1249–58. 10.3174/ajnr.A0612 17698524 PMC7977648

[20] Saindane AM Boddu SR Tong FC Dehkharghani S Dion JE. Contrast-enhanced time-resolved MRA for pre-angiographic evaluation of suspected spinal dural arterial venous fistulas. J Neurointerv Surg. (2015) 7:135–40. 10.1136/neurintsurg-2013-010981 24463440

